# Expression and function of CCN2-derived circRNAs in chondrocytes

**DOI:** 10.1007/s12079-023-00782-7

**Published:** 2023-09-11

**Authors:** Soma Kato, Kazumi Kawata, Takashi Nishida, Tomomi Mizukawa, Masaharu Takigawa, Seiji Iida, Satoshi Kubota

**Affiliations:** 1 Department of Biochemistry and Molecular Dentistry, Okayama University Graduate School of Medicine, Dentistry and Pharmaceutical Sciences, 2-5-1 Shikata-Cho, Kita-Ku, Okayama, 700-8525 Japan; 2 Department of Oral Maxillofacial Reconstructive Surgery, Okayama University Graduate School of Medicine, Dentistry and Pharmaceutical Sciences, Okayama, Japan; 3 Advanced Research Center for Oral and Craniofacial Sciences, Okayama University Faculty of Medicine, Dentistry and Pharmaceutical Sciences, Okayama, Japan

**Keywords:** Chondrocyte, *CCN2*, Circular RNA, *ACAN*, Chondrocytic differentiation

## Abstract

Cellular communication network factor 2 (CCN2) molecules promote endochondral ossification and articular cartilage regeneration, and circular RNAs (circRNAs), which arise from various genes and regulate gene expression by adsorbing miRNAs, are known to be synthesized from *CCN2* in human vascular endothelial cells and other types of cells. However, in chondrocytes, not only the function but also the presence of *CCN2*-derived circRNA remains completely unknown. In the present study, we investigated the expression and function of *CCN2*-derived circRNAs in chondrocytes. Amplicons smaller than those from known *CCN2*-derived circRNAs were observed using RT-PCR analysis that could specifically amplify *CCN2*-derived circRNAs in human chondrocytic HCS-2/8 cells. The nucleotide sequences of the PCR products indicated novel circRNAs in the HCS-2/8 cells that were different from known *CCN2*-derived circRNAs. Moreover, the expression of several *Ccn2*-derived circRNAs in murine chondroblastic ATDC5 cells was confirmed and observed to change alongside chondrocytic differentiation. Next, one of these circRNAs was knocked down in HCS-2/8 cells to investigate the function of the human *CCN2*-derived circRNA. As a result, *CCN2*-derived circRNA knockdown significantly reduced the expression of aggrecan mRNA and proteoglycan synthesis. Our data suggest that *CCN2*-derived circRNAs are expressed in chondrocytes and play a role in chondrogenic differentiation.

**Graphical abstract:**

**Figure:**
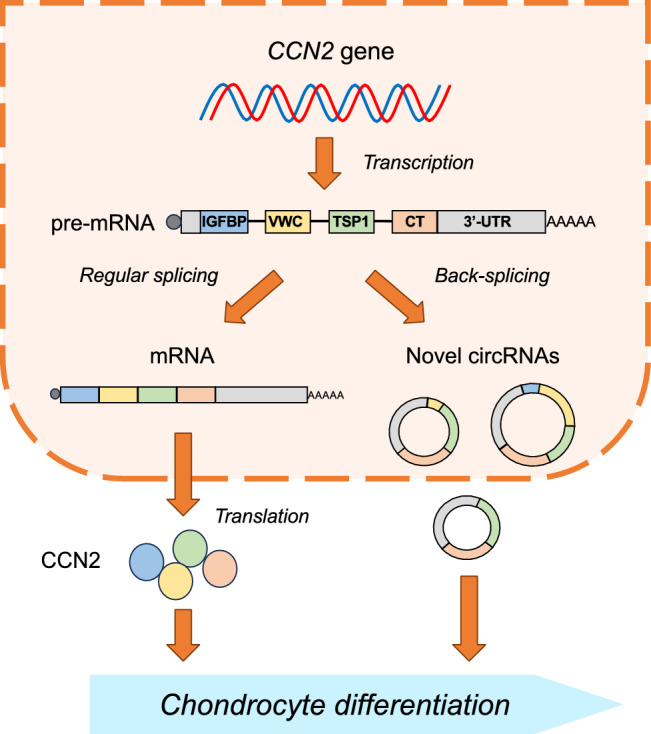
Production and role of CCN2-derived RNAs in chondrocytes

## Introduction

Cellular communication network factor 2 (CCN2) (Perbal et al. 2018) is a secreted protein expressed in various mesenchymal cells such as chondrocytes, fibroblasts, osteoblasts, and vascular endothelial cells (Chaqour 2020; Kubota and Takigawa 2011; Twigg 2018). In chondrocytes, CCN2 promotes cell proliferation and differentiation (Nakanishi et al. 2000; Nishida et al. 2017, 2008). CCN2 belongs to the CCN family, which consists of six members from CCN1 to CCN6. CCNs are composed of four modules starting from the N-terminal insulin-like growth factor binding protein-like module (IGFBP), followed by the von Willebrand factor type C module (VWC), the thrombospondin-1 type 1 repeat module (TSP1), and the C-terminal cystine knot module (CT) (Kubota and Takigawa 2011; Leask 2020; Perbal 2018). The mRNAs of CCNs are characterized by the retention of a long 3′-untranslated region (UTR). The half-lives and translation efficacies of these *CCN* mRNAs are controlled by the cis-elements in the 3′-UTR, typically miRNA targets (Kubota and Takigawa 2015). Structurally, *CCN5* is the only exception, in which the CT module is not encoded, and a short 3′-UTR is included (Lau 2016; Perbal 2013). In the case of CCN2, multiple miRNA targets are indicated in the 3′-UTR. Since CCN2 is highly expressed in a variety of tissues during development, the production and significant role of the circular RNAs (circRNAs) derived from *CCN2* in these tissues are suspected.

Circular RNAs were first reported in 1976 as viroids, the smallest plant pathogens (Sanger et al. 1976). Subsequently, various circRNAs were discovered in eukaryotic cells in several organisms including mammals (Capel et al. 1993; Ford and Ares 1994; Grabowski et al. 1981). Circular RNAs are generated via back-splicing during the maturation of linear pre-mRNA and forms rings containing one or more exons without poly-A tails (Chen and Yang 2015). Moreover, it is known that multiple circRNAs can be generated from a single pre-mRNA, such as RAN binding protein 17 (RANBP17) pre-mRNA, depending on the combination of exons and introns (Chen and Yang 2015; Szabo and Salzman 2016). The functions of circRNAs are diverse (Bi et al. 2018; Du et al. 2017, 2016). For example, circSMARCA5, which is the SWI/SNF-related, matrix-associated, actin-dependent regulator of chromatin subfamily A member 5-derived circRNA, performs RNA–DNA hybridization and transcription regulation (Conn et al. 2017; Xu et al. 2020). In contrast, ciRS-7 (hsa_circ0002484) functions as a miRNA sponge, binding to miRNAs and inhibiting their function (Memczak et al. 2013; Thomson and Dinger 2016; Zhong et al. 2019; Zou et al. 2019).

**Fig. 1:**
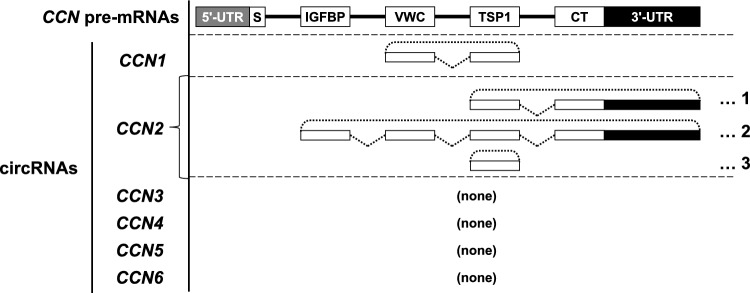
Structures of *CCN* pre-mRNAs and known *CCN*-derived circRNAs. The *CCN* pre-mRNAs contain 5′- and 3′- untranslated regions (UTRs) and four exons encoding characteristic protein modules: IGFBP (IGF-binding protein-like), VWC (von Willebrand factor type C), TSP1 (thrombospondin-1 type 1 repeat), and CT (C-terminal cystine knot). A small box with "S" indicates the region encoding signal peptides for secretion. Known *CCN1-*derived circRNA (circBase ID, hsa_circ_0114343; circBank ID: hsa_circCYR61_001) consists of VWC and TSP1 exons. Three types of *CCN2-*derived circRNAs are known. Each *CCN2-*derived circRNA structure consists of TSP1, CT, and 3′-UTR (1: circBase ID, hsa_circ_0077863; circBank ID: hsa_circCTGF_003), all of the module-encoding exons and 3′-UTR (2: circBase ID, hsa_circ_0077864; circBank ID, hsa_circCTGF_001), or a single TSP1 exon (3: circBase ID, hsa_circ_0077865; circBank ID: hsa_circCTGF_002). No circRNAs derived from *CCN3*, *CCN4*, *CCN5*, and *CCN6* have been discovered

Among the *CCNs*, no circRNAs produced from *CCN3-6* pre-mRNAs have been reported. On the other hand, *CCN1* and *2* have been shown to yield a few circRNAs*.* In particular, *CCN2*-derived circRNA is known to be expressed in human vascular endothelial cells (http://www.circbase.org) (Fig. 1). In chondrocytes, however, the biological functions or presence of such *CCN2*-derived circRNA remains unclear. In this study, we investigated the expression and biological function of *CCN2*-derived circRNAs in human and murine chondrocytic cells. Herein, we show that *CCN2*-derived circRNAs were expressed in chondrocytes, but their structures were different from those previously reported in other cell types (http://www.circbase.org). Furthermore, our results suggest that *CCN2*-derived circRNA plays a role in chondrocyte differentiation.

## Materials and methods

### Antibodies

For immunoblotting, anti-CTGF (CCN2) (Abcam, Cambridge, UK), β-actin (Fujifilm, Tokyo, Japan), anti-Aggrecan (Cosmo Bio Co, Tokyo, Japan), anti-SOX9 (Merck, Darmstadt, Germany), and anti-TypeII collagen (Proteintech, Rosemont, IL, USA), antibodies were employed. The secondary antibodies, horseradish peroxidase (HRP)-conjugated anti-rabbit IgG and anti-mouse IgG were purchased from Cell Signaling Technology (Danvers, MA, USA).

### Cell culture

Human chondrocytic cell line HCS-2/8 cells that stably retain mature chondrocytic phenotype and murine chondrocyte progenitor cell line ATDC5 cells were cultured in Dulbecco′s Modified Eagle’s Medium (DMEM) and alpha-modified Eagle’s minimum essential medium (αMEM) containing 10% fetal bovine serum (FBS), respectively. All cells were cultured at 37 °C in humidified air with 5% CO_2_. To induce ATDC5 cell differentiation, the culture medium was replaced with fresh αMEM containing 5% FBS and ITS (insulin–transferrin–selenium; Corning, Corning, NY, USA) every 3 days after the cells reached confluence.

### RNA extraction

HCS-2/8 cell RNA was harvested 3 days after cell seeding. ATDC5 cell RNA was harvested every 3 days from 0 to 21 days after differentiation induction. RNA extraction from HCS-2/8 and ATDC5 cells was performed with the RNeasy® Mini Kit (Qiagen, Hilden, Germany) according to the protocol provided by the manufacturer.

### Ribonuclease R treatment

To remove linear RNA from the extracted RNA, 1 μg of RNA was incubated with 0 U, 1 U, 5 U, and 10 U of Ribonuclease R (RNaseR; AR BROWN, Tokyo, Japan) in the attached reaction buffer (0.2 M Tris–HCl, 1 M KCl, and 1 mM MgCl_2_) at 37 °C for 15 min, followed by an inactivation process at 95 °C for 3 min.

### Reverse transcription (RT) polymerase chain reaction (PCR)

Five hundred nanograms of RNA was reverse-transcribed with PrimeScript cDNA synthesis kits (Takara, Japan). Thereafter, PCR was performed according to the protocol of Quick Taq HS DyeMix (Toyobo, Osaka, Japan)**.** The number of PCR cycles was within the range of 30–40 cycles in each experiment. For circRNA detection, primer sets were designed to specifically amplify the segments containing the back-splice junctions of human and murine* CCN2*-derived circRNAs (Table 1).

**Table 1:** Primers for amplifying *CCN2*- and *Ccn2*-drived circRNAs

Target gene (human)	Direction of primer	Recognized region	Sequence (5′→3′)	Length (Nucleotides)
*CCN2*-derived circRNA	BS	3′-UTR	GGA CAG CTT GTG GCA AGT GA	20
	BA	IGFBP	CAC GCC GAT CTT GCG GTT T	19
	BA	TSP1	ACC AGG CAG TTG GCT CTA ATC	21
*Ccn2*-derived circRNA	BS	3′-UTR	CTA GCG AGA GCT GAG CAT GT	20
	BA	TSP1	GTC TTA GAA CAG GCG CTC CA	20

BS, sense primer for detecting back-spliced product; BA, anti-sense primer for detecting back-spliced product

### Agarose gel electrophoresis

PCR products were separated via electrophoresis on 1.5% or 2% agarose gels with Tris–acetate–EDTA buffer containing ethidium bromide and visualized with a UV transilluminator (Kurabo, Osaka, Japan). The intensities of the visualized bands were quantified using the image processing software ImageJ (https://imagej.nih.gov/ij/).

### DNA sequencing

The bands containing the target DNA fragments were excised from the agarose gels. The DNA was purified according to the protocol of the QlAquick Gel Extraction Kit (Qiagen). The purified DNA was ligated into pGEM®-T Easy Vector (Promega, Madison, WI, USA) using the attached ligase mixture. Thereafter, plasmid DNA amplified with *Escherichia coli* was extracted using the GenEluteTM Plasmid Miniprep Kit (Merck). The nucleotide sequences of the plasmid DNA from the Sp6 and T7 primer binding sites were determined using a commercial service (FASMAC, Atsugi, Japan). The obtained nucleotide sequences were analyzed with the online tools and databases provided by the National Center for Biotechnology Information (NCBI: https://www.ncbi.nlm.nih.gov).

### RNAi

A 27-mer siRNA duplex containing 2 deoxynucleotides (5′-AAU AUU GUG UGU GUG ACG AGC CC dAdA-3′and 5′- UUG GGC UCG UCA CAC ACA CAA UAU UUA-3′) was designed for the specific knockdown of the *CCN2*-derived circRNA (A), targeting the back-splice junction. This siRNA duplex and a negative control siRNA were obtained from Nippon Gene (Tokyo, Japan). The siRNA duplex was transfected into HCS-2/8 cells (400,000 cells per well in 6-well plates) using siPORT™ NeoFX™ Transfection Agent (Thermo Fisher Scientific, Waltham, MA, USA) and incubated at 37 °C in humidified air with 5% CO_2_. After 24 h, the medium was replaced with a fresh one, and the cells were incubated for another 24 h. Thereafter, the RNAs were collected for subsequent analyses.

### Quantitative real-time PCR (qPCR)

The real-time quantitative monitoring of PCR amplification was performed using the SYBR Green Realtime PCR Master Mix (Toyobo) according to the manufacturer’s protocol. The fluorescent signals were detected and analyzed using the StepOnePlus™ Real-Time PCR System (Thermo Fisher Scientific). The primers used for the qPCR analysis are described in Table 2.

**Table 2:** Primers for real-time PCR

Target gene	Direction of primer	Sequence (5′→3′)	Length (Nucleotides)
*Gapdh*	S	CAC TCA CGG CAA ATT CAA CGG CA	23
	AS	GAC TCC ACG ACA TAC TCA GCA C	22
*Acan*	S	CCT CGG GCA GAA GAA AGA	18
	AS	GTC TCA TGC TCC GCT TCT GT	20
*Col2a1*	S	GGA ATT TGG TGT GGA CAT AGG G	22
	AS	GGT CAG GTC AGC CAT TCA GT	20
*Col10a1*	S	CCT GGT TCA TGG GAT GT	17
	AS	CCA GGA ATG CCT TGT TCT	18
*GAPDH*	S	GCC AAA AGG GTC ATC ATC TC	20
	AS	GTC TTC TGG GTG GCA GTG AT	20
*SOX9*	S	CAA CCA GAA TTC CCT TTG GA	20
	AS	TGC TCC ATT TAG CCA AGG TT	20
*ACAN*	S	TTC GGG CAG AAG AAG GAC	18
	AS	CGT GAG CTC CGC TTC TGT	18
*COL2A1*	S	GAG GGC AAT AGC AGG TTC ACG TA	23
	AS	TGG GTG CAA TGT CAA TGA TGG	21
*COL10A1*	S	GAG TAT GTC CAC TCC TCT T	19
	AS	CAT TCT TTT CAG CCT ACC TC	20
*CCN2*	S	GCA GGC TAG AGA AGC AGA GC	20
	AS	ATG TCT TCA TGC TGG TGC AG	20

S, sense primer; AS, anti-sense primer

### Immunoblotting

HCS-2/8 cells were lysed in a RIPA buffer (50 mM Tris–HCl, pH 8.0, 150 mM NaCl, 1% Igepal CA-630, 0.5% deoxycholate, and 0.1% sodium dodecyl sulfate: SDS). Aggrecan was processed by chondroitinase ABC 0.1 unit/mg CS (Merck, Darmstadt, Germany) in 50 mM Tris acetate, 10 mM EDTA, pH 7.6 for 1 h at 37 °C, followed by boiling at 95 °C for 5 min in 0.5% SDS, 20 mM dithiothreitol (Lark et al. 1995). The lysate diluted in 1 × SDS sample buffer (50 mM Tris–HCl, pH6.8, 2% SDS, 5% glycerol, 2% bromophenol blue, 5% 2-mercaptoethanol) was boiled at 95 °C for 3 min. The lysate was subjected to SDS–polyacrylamide gel electrophoresis in 6%, 8%, and 10% polyacrylamide gels. Proteins were transferred onto polyvinylidene difluoride membranes with a wet or semi-dry blotting apparatus. The membranes were then incubated for 1 h in a blocking buffer [5% non-fat milk in phosphate-buffered saline (PBS)] and subsequently incubated overnight at 4 °C with anti-CCN2 (1:1000), β-actin (1:1000), anti-Aggrecan (1:10), anti-SOX9 (1:500), and anti-TypeII collagen (1:400) antibodies in the blocking buffer. The membranes were incubated with HRP-conjugated anti-rabbit IgG (1:2000) and anti-mouse IgG (1:2000) antibodies in the blocking buffer at room temperature for 1 h.

### Glycosaminoglycans (GAG) assay

After 72 h of HCS-2/8 cell culture, the culture supernatants were collected. Cellular proteins were extracted by 2%Triton X-100/PBS. The lysate was treated at 65 °C for 90 min with 1 mg/ml actinase E in 0.2 M Tris–HCl (pH 7.5) and 5 mM CaCl_2_. Twenty microliters of the supernatant or cell lysate were added to 200 μl of 1,9 dimethylmethlyene blue (DMMB) reagent (38.46 μM DMMB, 40.5 mM glycine, 27.38 mM NaCl, 0.6% acetic acid). Thereafter, the absorbance was read using a plate reader at a wavelength of 525 nm immediately. GAG amount was normalized to that of the total protein. The total protein amount was measured with the BCA protein assay kit (Thermo Fisher Scientific) according to the manufacturer’s protocol.

### Luciferase reporter assay

The miR-181a reporter construct pGL3-181TS with a miR-181-5p target sequence in the *CCN1* mRNA 3′-UTR was produced in a previous study (Sumiyoshi et al. 2013) by using pGL3-L(+) vector containing a firefly luciferase gene (Kubota et al. 1999). HCS-2/8 cells were seeded in 12-well plates at approximately 2 × 10^5^ cells per well. Seven hundred nanograms of pGL3-181TS and 350 ng of phRL-TK (int-) (Promega, Madison, WI, USA) transfection was performed with X-tremeGENE 9 DNA transfection reagent (Merck) into HCS-2/8 cells transfected with si-circRNA (A) or control siRNA. After 48 h, luciferase activity was measured using the Dual-Luciferase Reporter Assay Kit (Promega). Firefly luciferase activity was normalized to *Renilla* luciferase activity.

### RNA analysis in silico

The secondary structure prediction of human CCN2 pre-mRNA was performed online with UNAfold (http://www.unafold.org). Putative miRNA binding sites were searched in silico on the TargetScan website (https://www.targetscan.org/vert_80/).

### Statistical analysis

The results are presented as mean ± standard deviation. The statistical significance of the differences between the mean values was determined with Dunnett’s test or Student’s t-test. Differences between the mean values were considered significant at a *P*-value of < 0.05.

## Results

### Circular RNAs were generated from CCN2 pre-mRNA in human chondrocytic HCS-2/8 cells

**Fig. 2:**
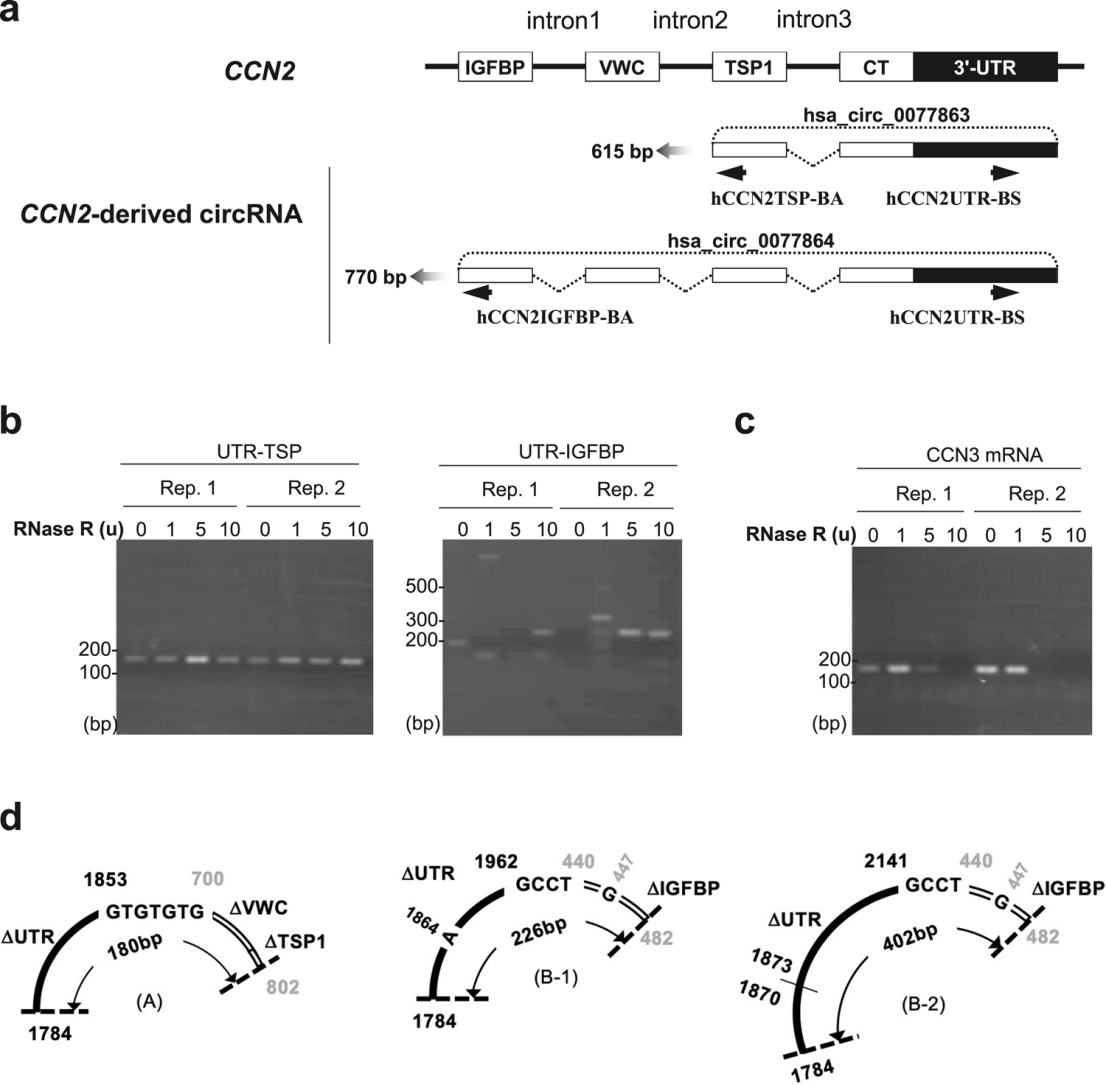
Detection of *CCN2*-derived circRNAs. **a** Positions of the primers designed in CCN2. The primer hCCN2TSP-BA antisense primer was designed on the TSP1 exon for the detection of a known *CCN2*-derived circRNA (hsa_circ_0077863), whereas hCCN2UTR-BS (sense primer) was designed on the UTR that is common between two *CCN2*-derived circRNAs (hsa_circ_0077863 and hsa_circ_0077864). The other hCCN2IGFBP-BA antisense primer was designed on the IGFBP exon to detect hsa_circ_0077864. **b** Detection of novel circRNAs in HCS-2/8 cells. RNAs of human chondrocytic HCS-2/8 cells were treated with 0 U (control), 1 U, 5 U, or 10 U of RNase R, followed by reverse transcription (RT) with random hexamers, PCR, and electrophoresis. The experiments were repeated twice, as indicated by Rep. 1 and Rep. 2. RT-PCR with hCCN2UTR-BS and hCCN2TSP-BA (UTR-TSP); a distinct band approximately 200 bp in length was stably observed regardless of the RNase R concentration. In contrast, with the primer set composed of hCCN2UTR-BS and hCCN2IGFBP-BA (UTR-IGFBP), bands were differentially observed in multiple positions. **c** Following the same protocol used for **b**, RT-PCR was performed to detect *CCN3* mRNA. The band from linear CCN3 mRNA was no longer detected with the 5U and 10U RNase R treatments. **d** Primary structures of the PCR products were determined using DNA sequencing. The sizes of the back-spliced products (A), (B-1), and (B-2) were 180 bp, 226 bp, and 402 bp, respectively. In CCN2-derived circRNA (A), the back-splice junction was located within the 5′-GTGTGTG-3′ sequence, which was flanked by the 700th-802nd sequence in the TSP1 exon and the 1784th-1853rd sequence of the 3′-UTR (numbers based on a human *CCN2* mRNA: GenBank accession: NM_001901.4). In the circRNAs (B-1) and (B-2), the back-splice junction was within the 5′-GCCT-3′ sequence, which was provided by either the IGFBP exon or the 3′-UTR. Point variations are also indicated

First, we examined whether circRNA was generated from *CCN2* pre-mRNA in human chondrocyte-like cell line HCS-2/8 cells. After HCS-2/8 cell RNAs were each treated with 0 U, 1 U, 5 U, and 10 U of RNase R to remove linear RNAs, RT-PCR was performed using antisense (*hCCN2*TSP-BA) and sense (*hCCN2*UTR-BS) primers (UTR-TSP set), which were designed in the TSP1 and 3′-UTR regions, respectively, to amplify the region surrounding the back-splice junction of a known *CCN2*-derived circRNA (circBase ID, hsa_circ_0077863; circBank ID: hsa_circ_CTGF_003) (Fig. 2a). The expected size of the PCR product using this primer to amplify this circRNA was 615 bp. The results demonstrate that the primer set yielded a distinct PCR product from the RNAs even after the treatment with high concentrations of RNase R (Fig. 2b). However, contrary to our expectation, the size of the PCR product was approximately 200 bp. In addition, RT-PCR was also performed using another antisense (*hCCN2*IGFBP-BA) primer and sense (*hCCN2*UTR-BS) primer (UTR-IGFBP set), which were designed in the IGFBP region and the 3′-UTR of a known *CCN2*-derived circRNA, respectively (circBase ID, hsa_circ_0077864; circBanc ID: hsa_circ_CTGF_001) (Fig. 2a). The expected size of the PCR product using this primer set to detect this particular circRNA was 770 bp. However, contrary to our expectation, two different amplicon sizes of approximately 250 bp and 400 bp were detected in the RNAs treated with high or low concentrations of RNase R, respectively (Fig. 2b). To confirm that RNase R was working effectively under these conditions, RT-PCR for *CCN3* mRNA was performed. The results show that bands of approximately 200 bp in length disappeared in the presence of 5 U and 10 U of RNase R (Fig. 2c). These results suggest that the PCR product in Fig. 2b is from RNase R-resistant *CCN2*-derived circRNA. Furthermore, the nucleotide sequence of the PCR product in Fig. 2b was determined by DNA sequencing**,** and the accurate size of the PCR product obtained with the UTR-TSP primer set (A in Fig. 2d) was 180 bp with a back-splice junction within the flanking “GTGTGTG” sequence (700th-802nd bases of VWC- and TSP1-encoding region and 1784th-1853rd bases of 3′-UTR in human *CCN2* mRNA: accession: NM_001901.4) (Fig. 2d). On the other hand, the sizes of the PCR products B-1 and B-2 (Fig. 2d) obtained with the UTR-IGFBP primer set were 226 bp and 402 bp, respectively. The nucleotide sequences of the PCR products B-1 and B-2 from *CCN2*-derived circRNAs exhibited back-splice junctions within the flanking “GCCT” sequences.

### Structural basis of CCN2 pre-mRNA that yields CCN2-derived circRNA (A)

**Fig. 3:**
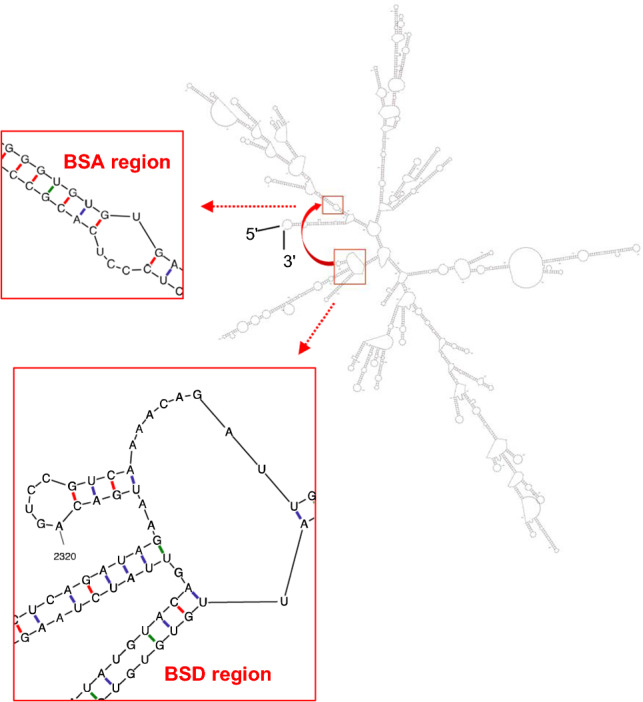
A predicted structure of the human *CCN2* mRNA. The 3′-segment of the *CCN2* pre-mRNA with 2400 bases, which contains both back-splicing donor (BSD) and acceptor (BSA) areas (red rectangles) of product A, was analyzed using UNAfold (http://www.unafold.org). Computed Gibbs free energy of this structure was -707.33 kcal/mol at 37 °C. The primary structures of BSD and BSA areas are made visible in the enlarged views of the boxed areas. Watson–Crick base pairing “G-C” and “A-U” are indicated with red and blue bars, respectively, whereas wobble “G-U” base pairing is shown in green. Note that these areas gathered in the stable secondary structure both contain unstable bulged structures, and a few hydrogen bonds between G and U around BSD may not have formed due to steric hindrance

To gain more insight into the mechanism that enables the back-splicing that forms the *CCN2*-derived circRNA (A), possible secondary structures of the CCN2 mRNA precursor were predicted employing the mFold server provided on the UNAfold website (http://www.unafold.org). Because of a current limitation of the system, the prediction of the entire structure of the full-length CCN2 pre-mRNA was not possible. However, the secondary structure prediction of a 2400-base RNA segment including the back-splicing sites revealed a stable secondary structure that could gather the back-splicing donor (BSD) and acceptor (BSA) nearby (Fig. 3). Interestingly, the BSD is located in an unstable portion of a stem-loop, in which G-U base paring is prevented due to conformational hindrance, facilitating the initiation of back-splicing events.

### Changes in Ccn2-derived circRNA expression levels with murine ATDC5 cell differentiation

**Fig. 4:**
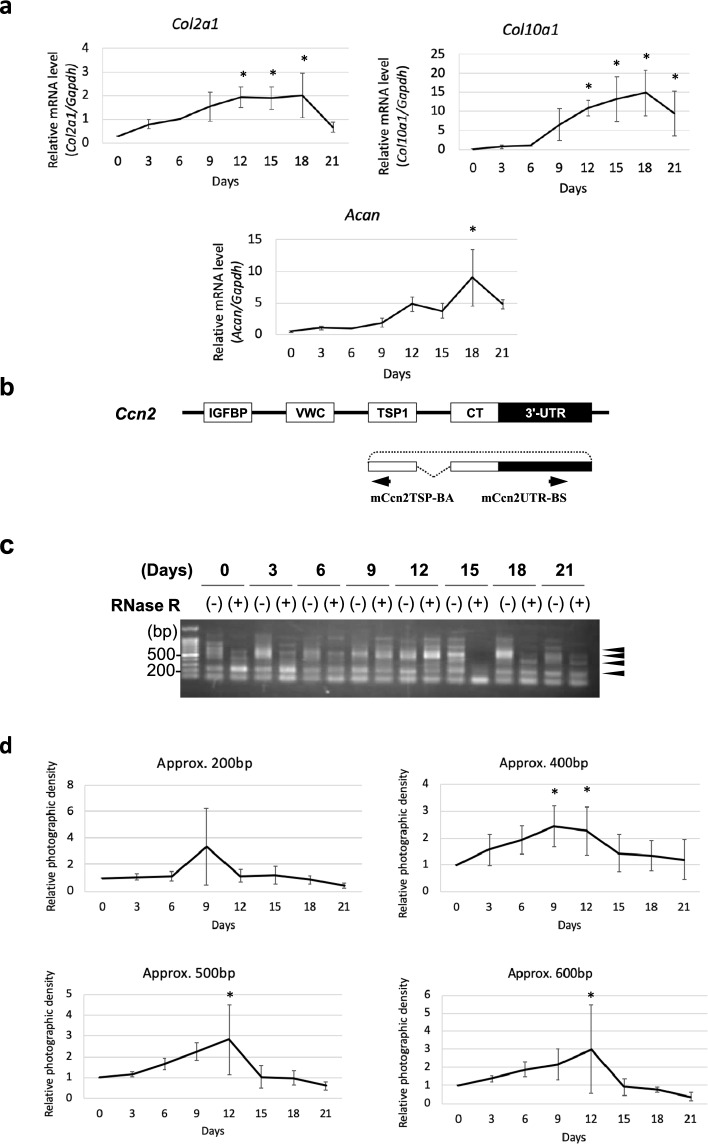
Changes in *Ccn2*-derived circRNA expression along with murine ATDC5 cell differentiation. **a** RNAs were collected from ATDC5 cells every 3 days during chondrocytic differentiation, and the expression levels of chondrocyte differentiation marker genes were measured with RT-qPCR. *Acan* and *Col2a1* expression levels increased from day 1 until day 18. *Col10a1* expression level increased gradually and was retained at higher levels until the end. All of the values were standardized against the expression level on day 0. RNAs from 3 independent cultures were analyzed. Asterisks represent significant differences at P < 0.05. **b** The primers were designed to specifically detect the back-spliced segments of the 3′-UTR and TSP1 exon in murine *Ccn2-*derived circRNAs. **c** Agarose gel electrophoresis after PCR using the primers in “b” showed RNase R-resistant, multiple bands. Four bands subjected to subsequent analysis are indicated with arrowheads **d** The photographic densities of bands at approximately 200, 400, 500, and 600 bp in “c (arrowheads)” were quantified using Image J. The bands of approximately 200 bp showed no significant changes. The intensities of the bands approximately 400 bp in length significantly increased 9 and 12 days after the induction of differentiation. The bands of approximately 500 and 600 bp displayed significantly higher intensities 12 days after the induction of differentiation. All of the values were standardized against the expression level on day 0. The values are presented as averages ± SD. *P < 0.05

To explore the function of *Ccn2*-derived circRNAs in chondrocyte differentiation, we employed a cell culture system with a murine chondrocyte progenitor cell line ATDC5 as a model system of chondrocyte differentiation. The chondrocytic differentiation of ATDC5 cells was observed by measuring the changes in the expression levels of chondrogenic differentiation marker genes via RT-qPCR (Fig. 4a). The results demonstrate that the expression levels of the type II collagen α1 chain gene (*Col2a1*) and aggrecan core protein gene (*Acan*)*,* which are typical marker genes of mature chondrocytes, increased until day 18 and then decreased until day 21. The expression levels of type X collagen α1 chain (*Col10a1*)*,* which is a terminal chondrocyte differentiation marker, increased along with chondrocyte differentiation and remained high until the end. Under these conditions, we investigated the expression of *Ccn2*-derived circRNAs. Unlike in human cells, no *Ccn2*-derived circRNAs have been reported in murine cells. Therefore, the antisense (*mCcn2*TSP-BA) and sense (*mCcn2*UTR-BS) primers used herein were designed in the TSP1-encoding region and 3′-UTR, respectively, so that they could detect possible orthologs of the known human *CCN2*-derived circRNA, hsa_circ_0077863 (Fig. 4b). Agarose gel electrophoresis after RT-PCR revealed multiple bands, including when the RNAs treated with 10 U of RNase R were used for amplification (Fig. 4c), which suggested the involvement of multiple circRNA species. Furthermore, quantitative analysis of the photographic density of each band indicated that the 400 bp signal significantly increased on day 9 and day 12, whereas the 500 and 600 bp signals peaked on day 12 (Fig. 4d), that is to say, the expression of the circRNAs producing these signals increased during the early steps of maturation in the chondrocytes. These results suggest that these circRNAs may play certain roles in chondrocyte differentiation.

### Structures of murine *Ccn2*-derived circRNAs

**Fig. 5:**
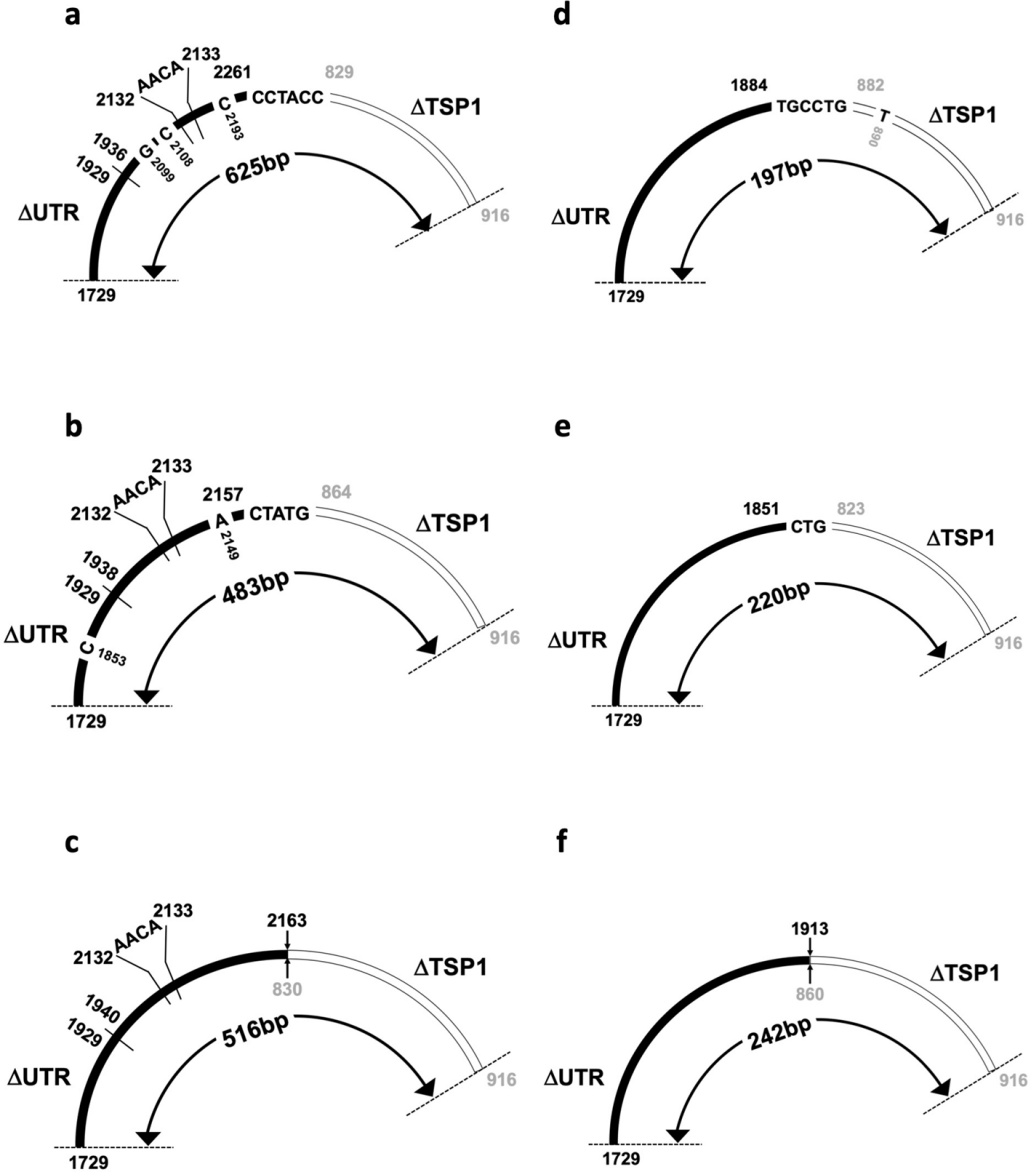
Structures of multiple murine *Ccn2*-derived circRNA segments around the back-splice junctions determined using DNA sequencing. The 625 bp (**a**), 483 bp (**b**), and 516 bp (**c**) PCR products contained an AACA insertion between the 2132nd and 2133rd bases in murine *Ccn2* cDNA (Accession: NM_010217.2). Additionally, the PCR products (**a**), (**b**), and (**c**) exhibited ATATAT (1930-1935th bases in murine *Ccn2* cDNA), ATATATAT (1930-1937th bases), and ATATATATAT (1930-1939th bases) deletion, respectively. Several PCR products (**a**), (**b**), (**d**: 197 bp), and (**e**: 220 bp) indicated that back-splicing occurred within the indicated 3–6 nucleotides flanking the TSP1 region or the 3′-UTR, whereas PCR products (**c**) and (**f**: 242 bp) showed distinct back-splice junctions between them. Point variations are also indicated

The nucleotide sequences of the murine PCR products were determined using Sanger DNA sequencing (Fig. 5). The results indicate that multiple novel circRNAs were present in murine chondrocytes. In contrast to the products detected in human cells, these PCR products were characterized by variations in the nucleotide sequences in comparison with that deposited in GenBank. The PCR products of 625 bp (Fig. 5a), 483 bp (Fig. 5b), and 516 bp (Fig. 5c) in length shared a common AACA insertion between the 2132nd and 2133rd bases in murine *Ccn2* mRNA (accession: NM_010217.2), suggesting a genetic variation. Moreover, these PCR products displayed ATATAT (1930-1935th bases), ATATATAT (1930-1937th bases), and ATATATATAT (1930-1939th bases) deletions in the same location, respectively, indicating instability in the dinucleotide repeats on the RNA. Regarding the back-splice junctions, the murine circRNAs showed more diversity. The back-splice junctions of several PCR products (Fig. 5a, b, d, and e) were located within the indicated 3–6 nucleotides. For example, in the case of Fig. 5f, back-splicing occurred within the trinucleotide that originated either from the 3′-end of the 3′-UTR or the 5′-end of the TSP1-encoding exon. In contrast with these four PCR products, the boundaries of the back-spliced exons were distinct in the other two amplicons (Fig. 5c and f).

### *CCN2*-derived circRNA knockdown in HCS-2/8 cells

**Fig. 6:**
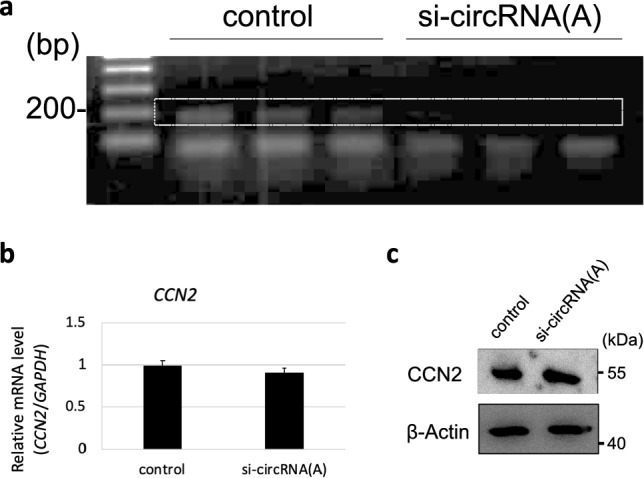
*CCN2*-derived circRNA (A) knockdown in HCS-2/8 cells. **a** Knockdown of *CCN2*-derived circRNA (A) was confirmed with RT-PCR via 3 independent sets of experiments. Specific signals of *CCN2*-derived circRNA (A) are indicated with a rectangle. **b** The expression level of *CCN2 *mRNA was measured using RT-qPCR. **c** The production level of CCN2 protein was examined by immunoblotting. Beta-actin was used as a loading control

To investigate the function of a *CCN2*-derived circRNA in chondrocyte differentiation in more detail, human *CCN2*-derived circRNA (A) was knocked down via RNA interference in HCS-2/8 cells (Fig. 6a). For this purpose, we designed and used a 27-mer siRNA duplex targeting the back-splice junction. This siRNA with two deoxynucleotides exerts high knockdown efficacy after being processed by the DICER complex. The RT-PCR results showed that *CCN2*-derived circRNA (A) was efficiently knocked down in HCS-2/8 cells (Fig. 6a). Importantly, no significant change was observed in the *CCN2* mRNA expression (Fig. 6b) and CCN2 protein production (Fig. 6c) levels upon the circRNA knockdown in HCS-2/8 cells. These results indicated that the circRNA-specific knockdown was successful.

### Effect of *CCN2*-derived circRNA knockdown on chondrocytic phenotype in HCS-2/8 cells

**Fig. 7:**
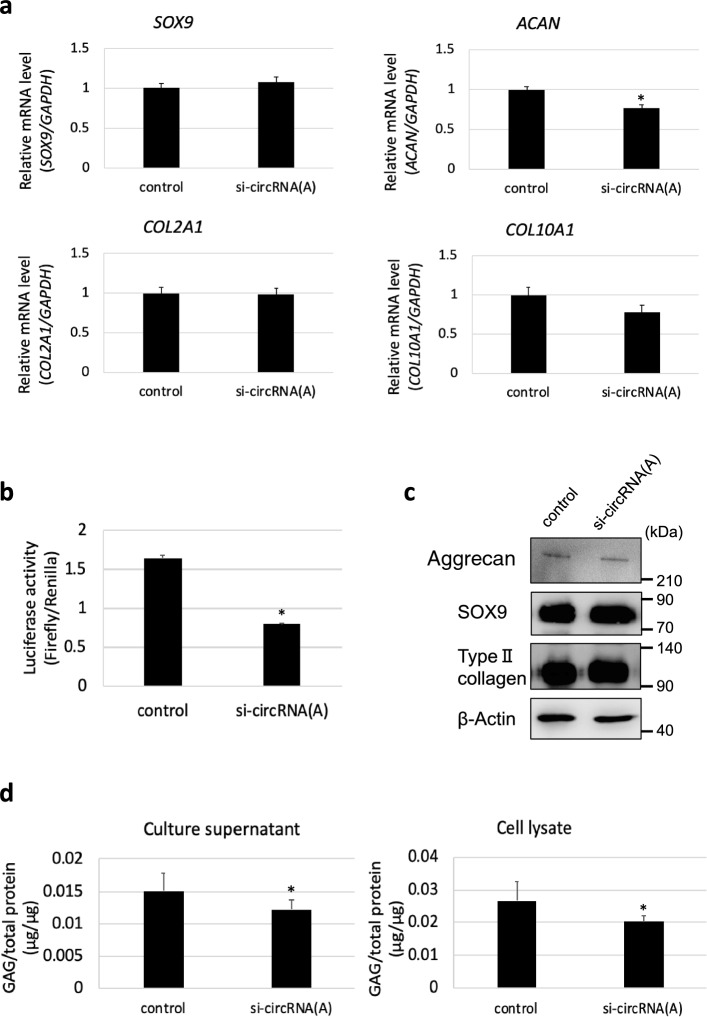
Effect of *CCN2*-derived circRNA (A) knockdown on chondrocytic phenotype of HCS-2/8 cells. **a** The expression level of *ACAN* mRNA significantly decreased in the *CCN2*-derived circRNA (A) knocked-down cells compared with the control. No significant differences were observed in the expression levels of *SOX9*, *COL2A1*, and *COL10A1*. The values are presented as the averages ± SD of 9 independent cultures. *P < 0.05. **b** Reporter gene assay was performed by using pGL3-181TS in control and *CCN2*-derived circRNA (A)-knocked-down HCS-2/8 cells. Luciferase activity was significantly reduced in *CCN2*-derived circRNA (A)-knocked-down HCS-2/8 cells. The values are presented as the averages ± SD of 3 independent cultures. *P < 0.05. **c** The production levels of aggrecan, SOX9, type II collagen proteins were examined by immunoblotting. Beta-actin was used as a loading control. **d** GAG assay was performed to measure the GAG levels in the culture supernatant and cell lysate obtained from *CCN2*-derived circRNA (A) knockeddown cells and the control cells. The values are presented as the averages ± SD of 6 independent cultures. *P < 0.05

Under the abovementioned conditions, the expression levels of the chondrocyte differentiation marker genes *ACAN*, *SOX9*, *COL2A*, and *COL10A* were evaluated using RT-qPCR (Fig. 7a). The results indicate that the expression level of *ACAN* mRNA significantly decreased in *CCN2*-derived circRNA-knocked-down HCS-2/8 cells compared with the control cells (Fig. 7a). However, there was no change in the expression levels of *SOX9*, *COL2A1*, and *COL10A1* (Fig. 7a). These results suggest that *CCN2*-derived circRNA (A) has a function in the regulation of *ACAN* mRNA expression specifically. Of note, this circRNA contains a miR-181-5p target in its 3′-UTR segment (Ling et al. 2020), and our previous study clearly showed that exogenous miR-181a-5p drastically decreased the *ACAN* mRNA in HCS-2/8 cells, strongly suggesting their direct interaction (Sumiyoshi et al. 2013). Therefore, to investigate the suppression mechanism of *ACAN* mRNA expression by knockdown of *CCN2*-derived circRNA (A), a reporter gene assay was performed using a firefly luciferase reporter that contains a target sequence of miR-181-5p (Sumiyoshi et al. 2013). The result of the reporter gene assay showed that luciferase activity was significantly decreased in *CCN2*-derived circRNA-knocked-down HCS-2/8 cells (Fig. 7b). These results suggest that *CCN2*-derived circRNA (A) enhances *ACAN* mRNA expression by the function as a miRNA sponge for miR-181-5p. At a protein level, no obvious difference was appreciable in the production of chondrocyte differentiation marker proteins by immunoblotting compared to the control (Fig. 7c). However, in the GAG assay, the amount of GAG in both culture supernatant and cell lysate was significantly reduced in *CCN2*-derived circRNA-knocked-down HCS-2/8 cells compared to the control cells (Fig. 7d). Considering that aggrecan is the major proteoglycan in cartilage and possesses tremendous amount of glycosaminoglycan chains, this result suggests that knockdown of the *CCN2*-derived circRNA (A) decreases aggrecan production.

## Discussion

*CCN2*-derived circRNAs are known to be expressed in several types of human cells including vascular endothelial cells, as revealed in previous studies (http://www.circbase.org). However, in chondrocytes, the presence of *CCN2*-derived circRNAs has not been reported. In this study, we revealed the expression of *CCN2*-derived circRNAs in human chondrocytic HCS-2/8 cells and murine chondroblastic ATDC5 cells. To date, no *Ccn2*-derived circRNAs have been detected in any murine cell. Therefore, this is the first study in which *Ccn2*-derived circRNAs have been discovered. Moreover, one of these *CCN2*-derived circRNAs was revealed to play a role in chondrocyte differentiation.

**Fig. 8:**
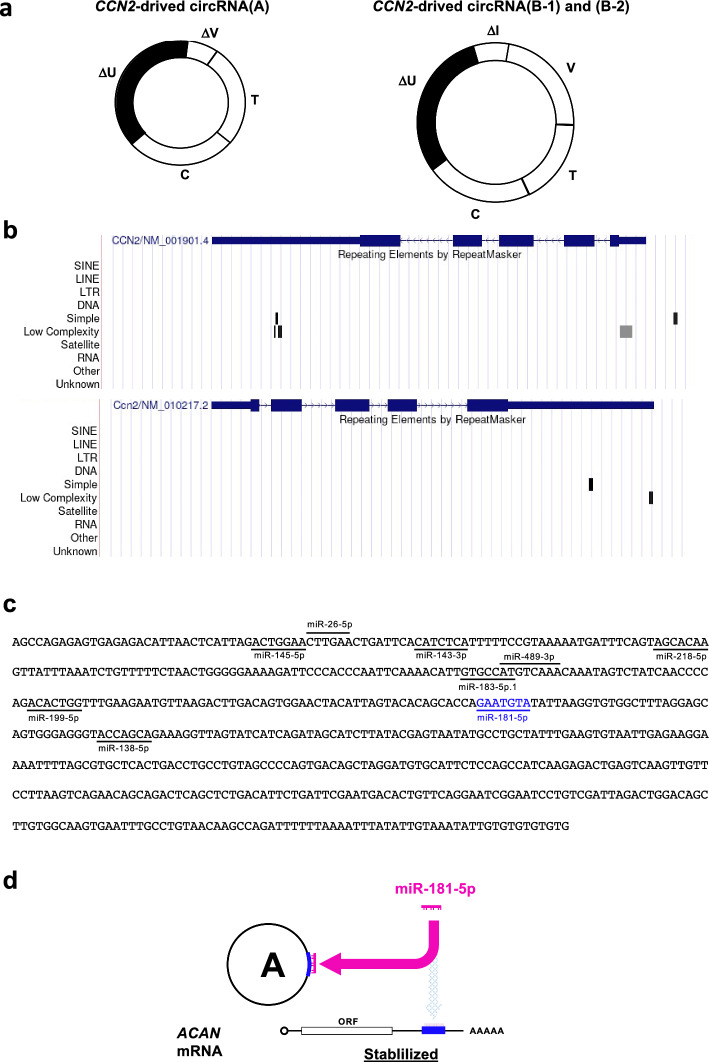
**a** Predicted structures of the novel human *CCN2*-derived circRNAs. *CCN2*-derived circRNA (A) was found to contain a VWC exon fragment (ΔV) as well as TSP1 (T)- and CT (C)-encoding regions with a 3′-UTR segment. *CCN2*-derived circRNAs (B-1) and (B-2) were predicted to consist of three module-encoding exons linked with 3′-UTR and IGFBP exon fragments (ΔU and ΔI). **b** Repetitive elements identified in the pre-mRNAs of human and murine CCN2 genes using RepeatMasker (https://www.repeatmasker.org). Track views of the distribution of repetitive elements in *CCN2* pre-mRNA (upper panel) and in *Ccn2* pre-mRNA (lower panel) were visualized on hg19 (chr 6: 132,268,517–132,273,319) and mm10 (chr10: 24,594,632–24,599,487) reference genomes, respectively, with the UCSC Genome Browser on the ENCODE portal site (https://www.encodeproject.org). Exons of the mRNAs are indicated at the top of each panel. Note that the 5′- and 3′-UTRs of *CCN2* contain a pair of low-complexity repetitive elements, which are absent in *Ccn2*. **c** Putative targets of miRNAs that are conserved in vertebrates in the 3′-UTR remnant of *CCN2*-derived circRNA (A). Target prediction was performed using TargetScan (https://www.targetscan.org/vert_80/). The common predicted target shared by human *ACAN* mRNA is shown in blue. Nucleotide sequences are displayed from 5′ to 3′ ends. **d** Possible mechanism of *ACAN* regulation by *CCN2*-derived circRNA (A). Sponging of miRNA-181-5p by the stable circRNA may result in increased *ACAN* expression at the post-transcriptional level

In our study, the identified portions of circRNAs contained only two exon fragments around the back-splice junction. However, considering them alongside the structure of the known circRNA (circBase ID, hsa_circ_0077863: circBank ID, hsa_circ_CTGF003), the novel *CCN2*-derived circRNA (A) is estimated to contain a 3′-UTR fragment, entire TSP1- and CT-module-encoding exons, and a fragment of the VWC-module-encoding exon (Fig. 8a). Similarly, the results suggest that the other two novel human *CCN2*-derived circRNAs (B-1) and (B-2) retain entire regions encoding the VWC, TSP1, and CT modules between the fragments of the IGFBP-encoding exon and the 3′-UTR, as observed in the other known circRNA (circBase ID, hsa_circ_0077864; circBank ID: hsa_circ_CTGF001 (Fig. 8a).

The biosynthetic pathway of these *CCN2*-derived circRNAs remains unclear. However, circRNAs are generally known to be produced from linear pre-mRNA, forming a circular structure via the fusion of a downstream 3′ back-splicing donor (BSD) with an upstream 5′ back-splicing acceptor (BSA) (Chen and Yang 2015). Although the mechanism of BSD–BSA fusion is poorly understood, several models, such as those of intron-pairing-driven cyclization (Zang et al. 2020) and cyclization carried out by RNA-binding proteins (RBPs) (Chen et al. 2021a, b), are currently being considered. In the intron-pairing-driven cyclization model, when a pair of inverse repetitive elements such as *Alu* elements, which are abundant in humans, or non-repetitive complementary sequences are present in adjacent introns, the downstream donor and upstream acceptor sites are drawn to each other, and cyclization occurs (Chen and Yang 2015; Ivanov et al. 2015; Zhang et al. 2016). The other “cyclization by RBPs” model proposes that RBPs bind to introns near the splice site and subsequently congregate the BSD and BSA (Conn et al. 2015). In our study, we discovered a functional *CCN2*-derived circRNA (A) that was stably expressed in human HCS-2/8 cells. According to the analysis using RepeatMasker (https://www.repeatmasker.org), the human CCN2 gene was found to contain no pairs of short interspersed nuclear elements (SINEs), such as *Alu* elements, which would facilitate back-splicing (Fig. 8b). Similarly, even fewer repetitive elements were identified in the murine CCN2 gene. However, the secondary structure formation analysis with mFold suggested that BSD and BSA could be gathered nearby in a stable structure formed in *CCN2* pre-mRNA. Therefore, we suspect that such a complex secondary structure enables the stable formation of *CCN2*-derived circRNA (A), probably with the assistance of particular RBPs stabilizing this structure. The other human CCN2-derived circRNA (B) and all of the murine *Ccn2*-derived circRNAs were found to be differentially expressed, suggesting that multiple circRNAs can be produced from the same pre-mRNA that is folded into multiple structures depending on the cellular conditions. As such, the production of *Ccn2*-derived circRNAs in ATDC 5 cells was regulated along with chondrocytic differentiation. RBPs are supposed to play significant roles in the selective stabilization of RNA structures therein.

Circular RNAs have various functions, one of which is acting as miRNA sponges (Hansen et al. 2013; Misir et al. 2022). Circular RNAs that function as miRNA sponges have miRNA binding sites in their nucleotide sequences. Therefore, such circRNAs can regulate target mRNA expression via the inhibition of miRNA activity by adsorbing miRNAs (Ebert et al. 2007). For example, ciRS-7, an oncogenic circRNA, plays an important role in the development and progression of various tumors by functioning as a miR-7 sponge (Chen et al. 2021a, b). In this study, human *CCN2*-derived circRNA (A) knockdown significantly decreased the expression level of *ACAN* mRNA, which is a chondrocyte differentiation marker gene that encodes aggrecan core protein (Fig. 7c). That is to say, *CCN2*-derived circRNA was found to promote the expression of the *ACAN* mRNA that supported the chondrocytic phenotype. Importantly, the 3′-UTR of *CCN2*-derived circRNA contains a binding site for miR-181-5p that could inhibit *ACAN* mRNA expression (Ling et al. 2020; Fig. 8c). Indeed, our present data indicated that binding of miR-181-5p to a target in the reporter mRNA was competed by this circRNA (Fig. 7). Therefore, *CCN2*-derived circRNA may inhibit miR-181-5p as a miRNA sponge and subsequently promote *ACAN* mRNA expression (Fig. 8d) by preventing its degradation. In contrast to *ACAN*, human *CCN2*-derived circRNA (A) did not alter the mRNA levels of *SOX9* and *COL2A1* (Fig. 7a). However, all of the three mRNAs were shown to be upregulated by a miR-181a-5p inhibitor in a previous study (Ye et al. 2022). In this study with chondrogenic mesenchymal stem cells, the effect of the miRNA inhibitor was the strongest on *ACAN* mRNA, which is consistent with our results. The sensitivity to a miRNA varies among target RNAs, and thus effect of the circRNA on miR-181-5p was detectable only through *ACAN* mRNA with the highest sensitivity (Fig. 7a). This idea also accounts for why CCN2 mRNA level was not affected by knocking down of the *CCN2*-derived circRNA. It should be also noted that although *ACAN* is a target of miR-181-5p in human chondrocytes, no miR-181-5p target is found in murine *Acan* mRNA by Targetscan. Considering together that circRNAs from human *CCN2* (Fig. 2) and those from murine *Ccn2* (Fig. 4) are structurally different, circRNAs are produced and regulating chondrocyte differentiation in a species-specific fashion. Further investigation is currently ongoing to clarify this issue.

In conclusion, this study revealed the expression of novel *CCN2*-derived circRNAs in human and murine chondrocytic cells. Moreover, the results indicate that these circRNAs play important roles in chondrocyte differentiation. Among chondrocyte markers, *ACAN* was the only one that was under the regulation of a particular circRNA. This finding raises the intriguing possibility that distinct circRNAs may regulate different chondrocyte differentiation markers. Therefore, the biological function of other *CCN2*-derived circRNAs should be further investigated in future research.
